# A Lack of GD3 Synthase Leads to Impaired Renal Expression of Connexins and Pannexin1 in *St8sia1* Knockout Mice

**DOI:** 10.3390/ijms23116237

**Published:** 2022-06-02

**Authors:** Diana Meter, Anita Racetin, Katarina Vukojević, Marta Balog, Vedrana Ivić, Milorad Zjalić, Marija Heffer, Natalija Filipović

**Affiliations:** 1 Department of Rheumatology and Clinical Immunology, University Hospital of Split, Spinčićeva 1, 21000 Split, Croatia; dianabajo53@gmail.com; 2 Laboratory for Early Human Development, Department of Anatomy, Histology and Embryology, University of Split School of Medicine, Šoltanska 2, 21000 Split, Croatia; anitamuic10@gmail.com (A.R.); kvukojev@gmail.com (K.V.); 3 Laboratory for Neurocardiology, Department of Anatomy, Histology and Embryology, University of Split School of Medicine, Šoltanska 2, 21000 Split, Croatia; 4 Department of Medical Biology and Genetics, Faculty of Medicine Osijek, Josip Juraj Strossmayer University of Osijek, Huttlerova 4, 31000 Osijek, Croatia; marthab007@gmail.com (M.B.); vedrana.ivic@mefos.hr (V.I.); marija.heffer@gmail.com (M.H.); 5 Department of Molecular Medicine and Biotechnology, Faculty of Medicine Rijeka, University of Rijeka, Branchetta brothers 20, 51000 Rijeka, Croatia; milzjalic@gmail.com

**Keywords:** kidney, GD3 synthase, *St8sia1*, connexins, pannexin 1

## Abstract

The aim of this study was to determine the effects of altered ganglioside composition on the expression of Cx37, Cx40, Cx43, Cx45, and Panx1 in different kidney regions of *St8sia1* gene knockout mice (*St8sia1* KO) lacking the GD3 synthase enzyme. Experiments were performed in twelve male 6-month-old mice: four wild-type (C57BL/6-type, WT) and eight *St8sia1* KO mice. After euthanasia, kidney tissue was harvested, embedded in paraffin wax, and processed for immunohistochemistry. The expression of connexins and Panx1 was determined in different regions of the kidney: cortex (CTX.), outer stripe of outer medulla (O.S.), inner stripe of outer medulla (IN.S.), and inner medulla (IN.MED.). We determined significantly lower expression of Cx37, Cx40, Cx45, and Panx1 in different parts of the kidneys of *St8sia1* KO mice compared with WT. The most consistent decrease was found in the O.S. where all markers (Cx 37, 40, 45 and Panx1) were disrupted in St8si1 KO mice. In the CTX. region, we observed decrease in the expression of Cx37, Cx45, and Panx1, while reduced expression of Cx37 and Panx1 was more specific to IN.S. The results of the present study suggest that deficiency of GD3 synthase in *St8sia1* KO mice leads to disruption of renal Cx expression, which is probably related to alteration of ganglioside composition.

## 1. Introduction

Connexins (Cx) are a family of transmembrane proteins that form gap junctions, critical for intercellular communication in many organs, including the kidney. The family of these proteins consists of more than 20 isoforms in humans and rodents, with marked homology between the different Cx in humans, rats, and mice [1]. The individual Cx are usually described by their molecular mass (for example, Cx43 has a molecular mass of 43 kDa) [1,2]. Six Cx oligomerize to form a ring-shaped hemichannel, the connexon, which docks to a connexon from an adjacent cell and forms a channel with a central aqueous pore [3]. The connexon hexamer can consist of one or more Cx isoforms, which are referred to as homomeric or heteromeric connexon accordingly [4]. Gap junctions are formed directly between adjacent cells and allow the transfer of small molecules and ions [1]. The ability to regulate Cx expression and function is important because gap junctions play a role in many physiological and pathophysiological processes, such as cell growth or hyperplasia, tissue differentiation, carcinogenesis, and hormone secretion and action [5,6,7,8]. Cx exist either within junctional plaques or as unopposed hemichannels in the plasma membrane. The plaques differ structurally and in composition from the surrounding plasma membrane [9,10,11]. Nonjunctional Cx channels in the plasma membrane are associated with lipid rafts, specifically organized microdomains enriched in cholesterol and glycosphingolipids [12,13]. Some Cx channels are also localized in caveolae, a subpopulation of lipid rafts that differ from other types by an invaginated morphology and the monotopic cholesterol-binding protein caveolin-1 [14,15,16]. Recent evidence revealed that the distribution of Cx channels differs in rafts with different lipid compositions. Moreover, specific biophysical properties of rafts might influence Cx channel function [17]. In addition to forming gap junctions, connexon hemichannels also provide a pathway for the release of ATP, glutamate, NAD+ and prostaglandin E2 from cells, which then act as paracrine messengers [18]. Cx are important for the normal development and physiology of a wide range of organs, including the nervous system, cardiovascular system, reproductive organs, and skin [19,20,21]. There are nine different isoforms of Cx in the kidneys, and their expression varies in different regions. They play a functional role in several regulatory mechanisms in the kidney, including regulation of renin secretion, tubuloglomerular feedback, and salt and water reabsorption. At the systemic level, renal Cx may contribute to the regulation of blood pressure and be involved in the pathogenesis of hypertension and diabetes [1].

Structurally similar to connexins are pannexins (Panx1, 2, and 3), hexameric membrane channels, which, unlike the Cx, mostly do not form gap junctions. While Panx2 is mainly expressed in the brain, Panx3 is widespread in many tissues, as well as Panx1, expressed also in the endothelium of renal vasculature and tubular segments [22]. Panx1 channels control the release of ATP and other nucleotides from many cell types, participate in apoptosis, and their activation is known for initiation of paracrine signaling that controls blood vessel constriction [23,24,25,26]. In addition to its importance in blood pressure control, there is also evidence about the role of Panx1 in the pathophysiology of insulin resistance and DM [27]. 

Important membrane components involved in cell signaling are gangliosides, the membrane sialic acid-containing glycosphingolipids that are ubiquitously found in tissues and are particularly abundantly expressed in the mammalian central nervous system [28]. They interact with other membrane components, with molecules of adjacent membranes and the extracellular matrix. Therefore, they are involved in other vital cellular processes such as cell growth, cell-to-cell recognition, cell adhesion, signal transduction, proliferation, differentiation, and autophagy [29,30,31,32]. Gangliosides are not evenly distributed in the membrane, but they are clustered in lipid rafts [12,13]. Ganglio- series glycosphingolipids are classified into a sialo-, a-series, b-series, and c-series gangliosides, depending on whether they have 0, 1, 2, or 3 sialic acid residues attached to the inner galactose residue. Ganglioside synthesis involves stepwise action of various glycosyl and sialyltransferases [30,33,34]. Blockage of ganglioside biosynthetic pathways at a particular step in synthesis results in a change in the ganglioside profile, which can cause deficiency and/or accumulation of certain gangliosides depending on the blocked site in synthesis [35]. Therefore, changes in ganglioside composition, ganglioside modifications, and the presence or absence of certain ganglioside species can lead to alterations in cellular metabolism. By selectively knocking down certain enzymes of the biosynthetic pathway in mice, the effects of altered ganglioside composition can be analyzed. For example, mice lacking the *St8sia1* gene encoding α-N-acetylneuraminate-α-2,8-sialyltransferase (EC 2.4.99.8; *St8sia1* knockout mice [KO]) lack the enzyme GD3 synthase, which catalyzes the transfer of sialic acid to GM3. This deficiency leads to accumulation of a-series gangliosides (GM1, GM2, GM3, GD1a) and a deficiency of b- and c-series gangliosides (GD3, GD1b, GT1b, GT1c, GT2, GT3) [36]. Loss of certain gangliosides markedly alters ganglioside composition, even though the total concentration of gangliosides in the KO mice remains the same as in wild-type mice (WT) [35]. 

GM3 ganglioside, which accumulates in GD3 synthase-deficient mice, is a dominant ganglioside that accounts for three-quarters of the total gangliosides present in the kidney [37]. It is particularly abundant in renal tissue and is thought to be involved in maintaining the charge-selective filtration barrier of the glomeruli via regulation of podocyte function [38]. The abundance of GM3 in podocytes suggests its role in the pathophysiology of glomerulopathies [39]. There is increasing evidence that GM3 has negative modulatory effects on insulin-mediated signaling pathways and is involved in the cascade leading to diabetic nephropathy [40,41,42,43]. In diabetes, the level of advanced glycation end products (AGE) is usually elevated and seems to be responsible for an increased concentration of a-series gangliosides in tissues, especially GM3, but also GM2 and GM1 [44]. Considering that GM3 plays an important role in maintaining the charge-selective filtration barrier of glomeruli, its altered expression may be an important factor in the deterioration of renal function in the pathogenesis of diabetic nephropathy [38,45]. It has been suggested that the accumulation of GM3 in hyperglycemia leads to depletion of functional renal parenchyma through activities that include clustering of gangliosides in lipid rafts [40]. Considering all this, the disruption of ganglioside composition in the kidney of *St8sia1* KO mice may be a good model for the alteration of ganglioside composition and properties of lipid rafts under diabetic conditions.

Considering that altered ganglioside composition affects the distribution of proteins in lipid rafts of different cell membranes and in this way probably influences the distribution, transport, and function of different Cx isoforms, the aim of this study was to determine the effects of specifically altered ganglioside composition on the expression of Cx37, Cx40, Cx43, Cx45, and Panx1 in different parts of the kidney of *St8sia1* KO mice. 

## 2. Results

### 2.1. Expression of Connexin 37 in Kidney of Wild Type and St8sia1-KO Mice

**Figure 1:**
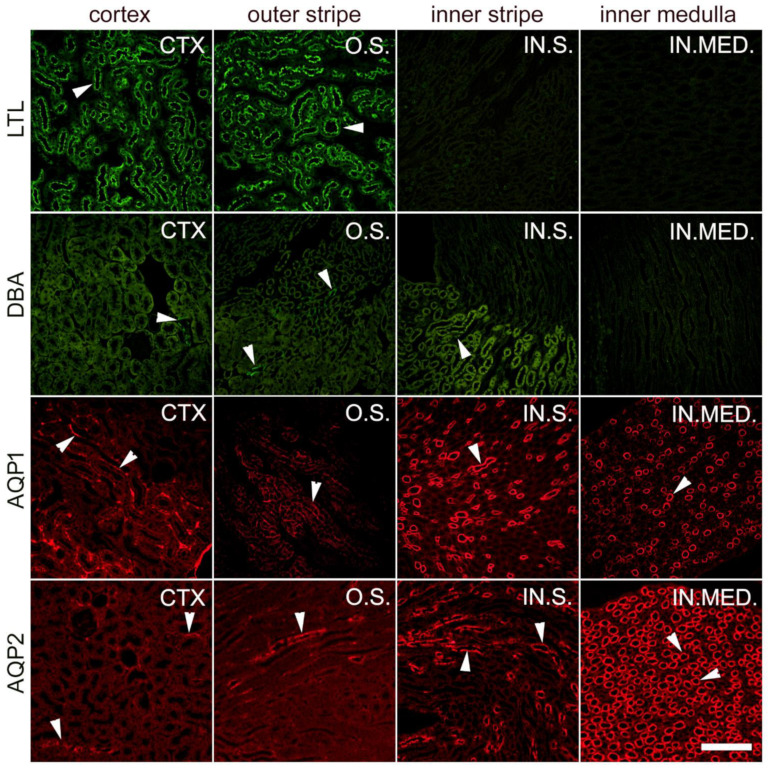
Distribution of tubular segment markers in different parts of the mice kidney. CTX.—cortex; O.S.—outer stripe of outer medulla; IN.S.—inner stripe of outer medulla; IN.MED.—inner medulla. Binding of *Lotus tetragonolobus* lectin (LTL) was specific to proximal tubules located in the cortex and outer stripe of the outer medulla (green; first row). Binding of *Dolichos biflorus* agglutinin (DBA) was found specifically in the distal tubules located in the cortex and in the outer and inner strips of the outer medulla (green; second row). The antibody against aquaporin 1 (AQP1) stained the proximal tubules in the cortex and outer stripe and the thin descending loop of Henle in the inner stripe and inner medulla (red; third row), whereas anti-aquaporin 2 (AQP2) stained various segments of the collecting ducts in the cortex, outer medulla, and inner medulla (red; fourth row). Scale-bar = 40 µm, refers to all (magnification 200×).

**Figure 2:**
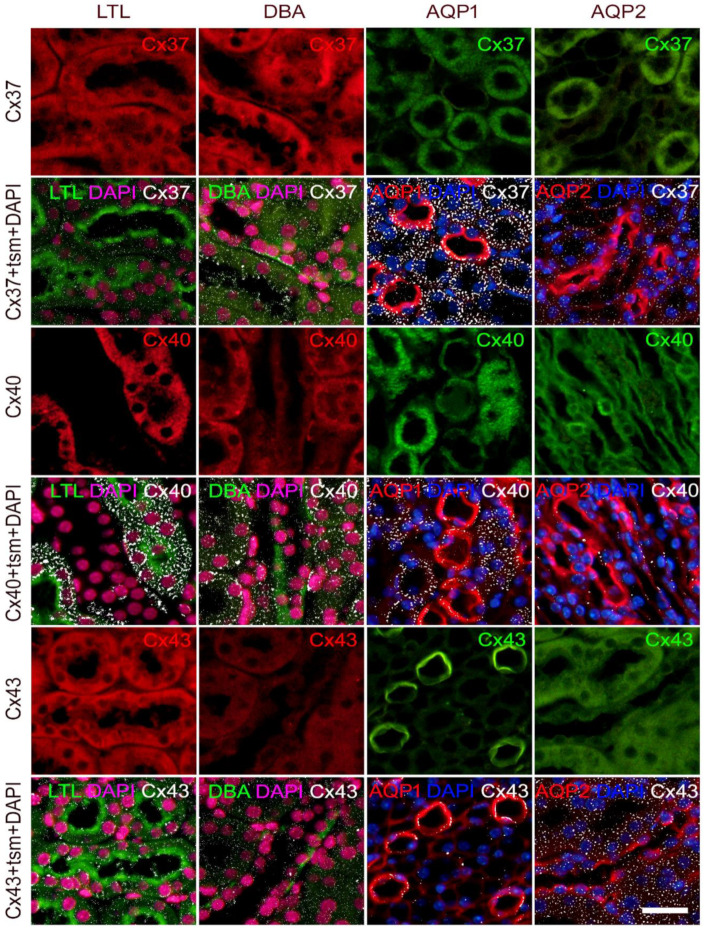
Colocalization of connexins 37, 40, and 43 with different tubular segment markers in the mice kidney. Sections from the wild-type mice. *Lotus tetragonolobus* lectin (LTL), proximal tubule marker (green; first column); *Dolichos biflorus* agglutinin (DBA), distal tubule marker (green; second column); aquaporin 1 (AQP1), thin descending loop of Henle marker (red; third column); aquaporin 2 (AQP2), collecting duct marker (red; fourth column). Nuclei were stained with DAPI (blue in third and fourth column; magenta—pseudocolorized in first and second column); tsm—tubular segment marker. Scale-bar = 20 µm, refers to all (magnification 400×).

**Figure 3:**
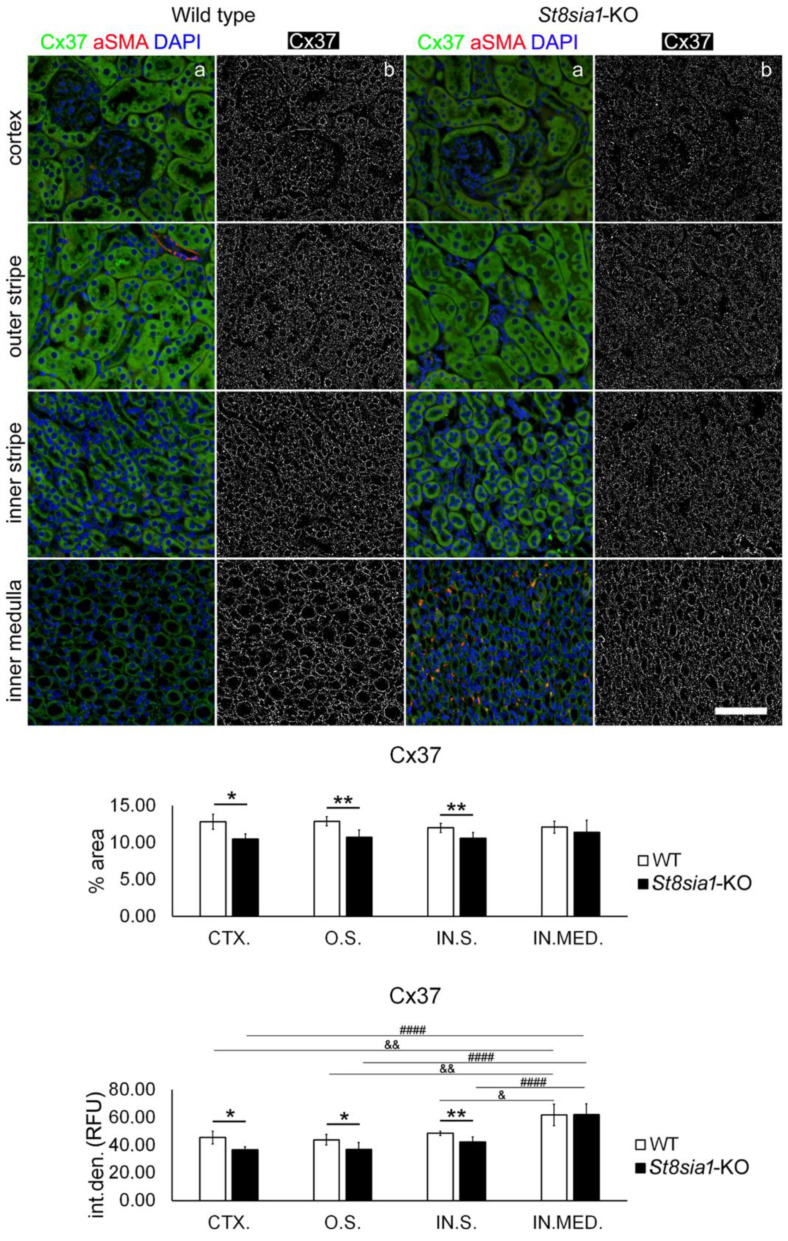
Expression of connexin 37 in different renal parts of the wild type and *St8sia1*-KO mice. WT—wild-type mice; *St8sia1*-KO—*St8sia1* knockout mice; CTX.—Cortex; O.S.—outer stripe of outer medulla; IN.S.—inner stripe of outer medulla; IN.MED.—inner medulla; % area—percentage of the section area occupied by Cx37 immunofluorescence; int.den. (RFU)—integrated density (relative fluorescence units). Column a—representative photomicrographs of the indicated kidney section areas stained with Cx37 (green) and alpha smooth actin (aSMA, red) antibodies; cell nuclei are stained blue. Column b—(same section as a) processed and thresholded photomicrographs prepared for analysis (inverted black for white on tresholded green image). Statistically significant difference between WT and *St8sia1*-KO: * *p* < 0.05; ** *p* < 0.01 (*t*-test for unequal variances). Comparison between different kidney areas in the same group—WT: & *p* < 0.05; && *p* < 0.01; or *St8Sia1*-#### *p* < 0.0001 (one-way ANOVA followed by Tukey’s test for dependent samples). Scale-bar = 20 µm, refers to all (magnification 400×).

Distribution of different tubular segment markers that we used to localize expression of Cx in different parts of the mice kidney are presented in Figure 1. Expression of Cx37 in mouse kidney was found to be abundant in all tubular structures in the cortex and inner and outer medulla and glomeruli of both WT and *St8sia1*-KO mice (Figure 2 and Figure 3). Double fluorescent staining with *Lotus tetragonolobus* lectin (LTL) showed the presence of Cx37 in the epithelial cells of proximal tubules, whereas coexpression with the distal tubule marker—*Dolichos biflorus* agglutinin (DBA), revealed its presence in the epithelium of distal tubules. In the medulla, double fluorescent staining with aquaporin 1 (AQP1) showed the strongest expression of Cx37 in an AQP1-immunoreactive epithelium of the descending thin limb of the loop of Henle and in an AQP1-negative epithelium of the thick ascending limb, whereas it was rarely present in the aquaporin 2 (AQP2) immunoreactive cells of the collecting ducts (Figure 2). Analysis revealed significantly lower Cx37 area (*p* = 0.0110) and integrated density (I.D.; *p* = 0.0231) in the CTX.; O.S. (% area *p* = 0.0012; I.D. *p* = 0.0273) and in the IN.S. (% area *p* = 0.0097; I.D. *p* = 0.0018) in the *St8sia1*-KO mice, compared with the WT (Figure 3). In WT mice, the highest Cx37 I.D. was found in IN.MED. It was significantly higher compared with CTX (*p* = 0.0084), O.S. (*p* = 0.0048), and IN.S. (*p* = 0.0274). The same was true for *St8sia1*- KO mice (*p* < 0.0001 for all three corresponding comparisons).

### 2.2. Expression of Connexin 40 in the Kidney of Wild-Type and St8sia1-KO Mice

**Figure 4:**
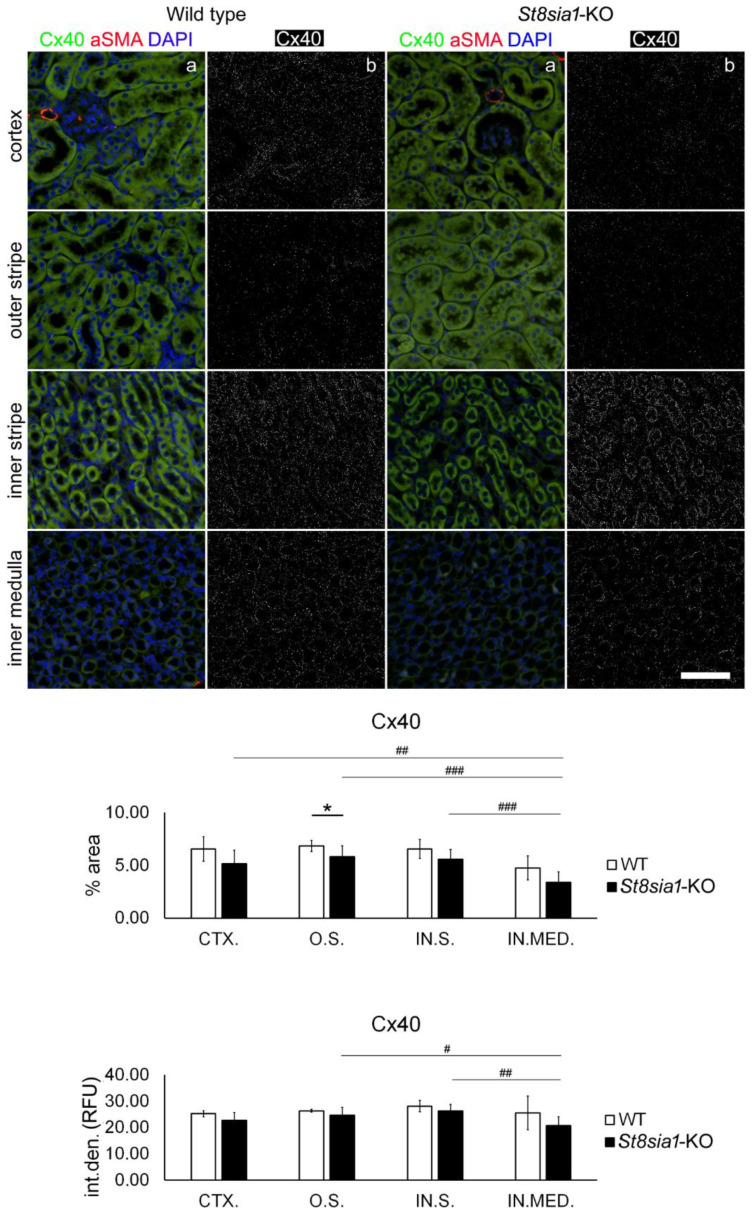
Expression of connexin 40 in different renal parts of the wild type and *St8sia1*-KO mice. WT—wild-type mice; *St8sia1*-KO—*St8sia1* knockout mice; CTX.—cortex; O.S.—outer stripe of outer medulla; IN.S.—inner stripe of outer medulla; IN.MED.—inner medulla; % area—percentage of sectional area occupied by Cx40 immunofluorescence; int.den. (RFU)—integrated density (relative fluorescence units). Column a—representative photomicrographs of the indicated kidney section areas stained with Cx40 (green) and alpha-smooth actin (red) antibodies; cell nuclei are stained in blue. Column b—(same section as a) processed and thresholded photomicrographs prepared for analysis (inverted black for white on thresholded green image). Statistically significant difference between WT and *St8sia1*- KO: * *p* < 0.05 (*t*-test for unequal variances). Comparison between different kidney areas in the same group: WT—there was no significant difference; for *St8Sia1*-# *p* < 0.05, ## *p* < 0.01, ### *p* < 0.001 (one-way ANOVA followed by Tukey’s test for dependent samples). Scale-bar = 20 µm, refers to all (magnification 400×).

Renal expression of Cx40 was found in all tubular structures in the cortex and inner and outer medulla, as well as in the glomeruli of both strains of mice, but Cx40 immunoreactive puncta did not appear so dense as Cx37 immunoreactive puncta (Figure 4). Direct comparison should be made with caution, since two different antibodies with different properties were used. That was the reason why we avoided to compare directly different connexins, although different densities (15 in comparison to 5% of area) might be observed from the graphical presentations. The most abundant expression of Cx40 was found in LTL-positive proximal tubule cells (PTCs) in the cortex and in AQP1-non-immunoreactive cells of the thick ascending limb in the medulla; it was also present at lower levels in DBA-positive distal tubule cells (DTCs) and AQP1-immunoreactive cells of the thin descending limb. Rarer but distinct Cx37 dotted patterns were clearly found in AQP2-immunoreactive cells of the collecting ducts (Figure 2). Quantification revealed significantly lower Cx40% area (*p* = 0.0489) in the O.S. of *St8sia1*-KO mice compared with WT. In WT mice, there was no significant difference in Cx40% area or I.D. between the kidney parts studied. However, in *St8sia1*-KO mice, the lowest Cx40% area was found in the IN.MED. which was significantly lower compared with CTX. (*p* = 0.0014), O.S. (*p* < 0.0001), and IN.S. (*p* = 0.0002). The Cx40 I.D. in IN.MED. was significantly lower compared with O.S. (*p* = 0.0124) and IN.S. (*p* = 0.0012). 

### 2.3. Expression of Connexin 43 in the Kidney of Wild-Type and St8sia1-KO Mice

**Figure 5:**
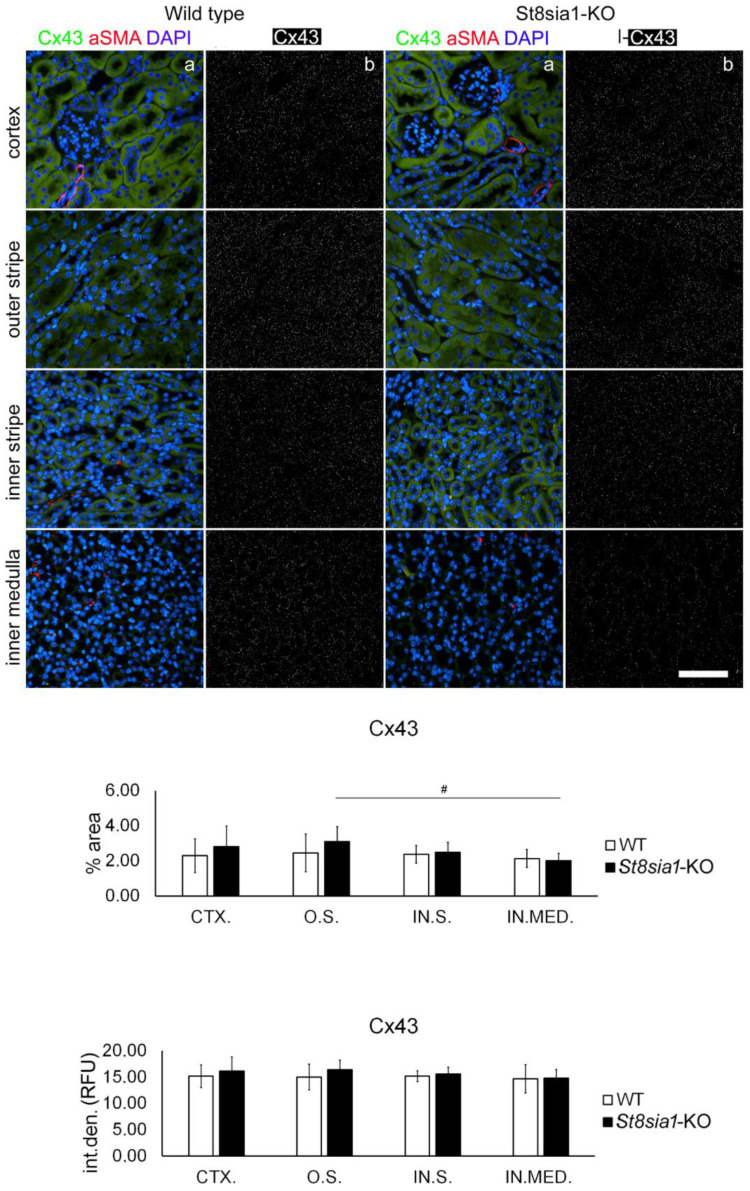
Expression of connexin 43 in different renal parts of the wild type and *St8sia1*-KO mice. WT—wild-type mice; *St8sia1*-KO—*St8sia1* knockout mice; CTX.—Cortex; O.S.—outer stripe of outer medulla; IN.S.—inner stripe of outer medulla; IN.MED.—inner medulla; % area—percentage of sectional area occupied by Cx43 immunofluorescence; int.den. (RFU)—integrated density (relative fluorescence units). Column a—representative photomicrographs of the indicated kidney sections stained with Cx43 (green) and alpha-smooth actin (red) antibodies; nuclei are stained in blue. Column b—(same section as a) processed and thresholded photomicrographs prepared for analysis (inverted black for white on thresholded green image). There was no significant difference in Cx43 expression between WT and *St8sia1*-KO. Comparison between different kidney areas in the same group WT—there was no significant difference; for *St8Sia1*-# *p* < 0.05 (one-way ANOVA followed by Tukey’s test for dependent samples). Scale-bar = 20 µm, refers to all (magnification 400×).

Although Cx43 was the least abundant compared with all other connexins examined, it was expressed in most tubular structures in the cortex and inner and outer medulla and glomeruli of both strains of mice, but it was scarcely present compared with all other connexins examined (Figure 5). Double immunohistochemistry revealed Cx43 expression in PTCs, DTCs, and APQ2-immunoreactive cells of collecting ducts. The clearest Cx43 presence was found in AQP1-immunoreactive thin descending limbs, whereas in contrast to all other connexins and Px1 examined, Cx43-immunoreactive puncta were rare (if not absent) in AQP1-nonimmunoreactive cells of the thick ascending limb (Figure 2). There were no significant differences in Cx43% area or I.D. between *St8sia1*-KO and WT mice in any of the renal areas examined. In WT mice, there was no significant difference in Cx43% area or I.D. between the kidney areas studied. However, in *St8sia1*-KO mice, the lowest Cx43% area was found in the IN.MED. which was significantly lower compared with O.S. (*p* = 0.01101), but there was no significant difference between the different kidney section areas when I.D. was compared. 

### 2.4. Expression of Connexin 45 in the Kidney of Wild-Type and St8sia1-KO Mice

**Figure 6:**
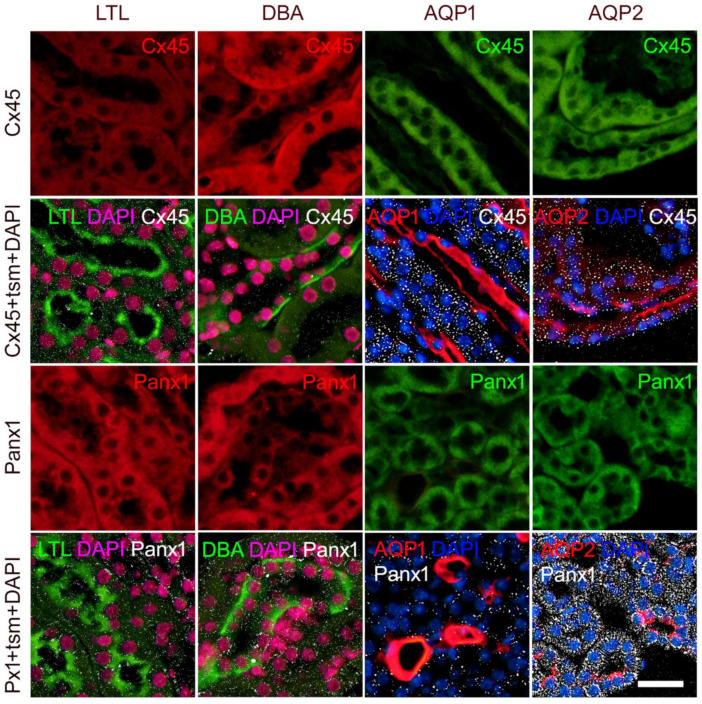
Colocalization of connexin 45 and Panx1 with different tubular segment markers in the mice kidney. Sections from the wild-type mice. *Lotus tetragonolobus* lectin (LTL), proximal tubule marker (green; first column); *Dolichos biflorus* agglutinin (DBA), distal tubule marker (green; second column); aquaporin 1 (AQP1), thin descending loop of Henle marker (red; third column); aquaporin 2 (AQP2), collecting duct marker (red; fourth column). Nuclei were stained with DAPI (blue in third and fourth column; magenta—pseudocolorized in first and second column); tsm—tubular segment marker. Scale-bar = 20 µm, refers to all (magnification 400×).

**Figure 7:**
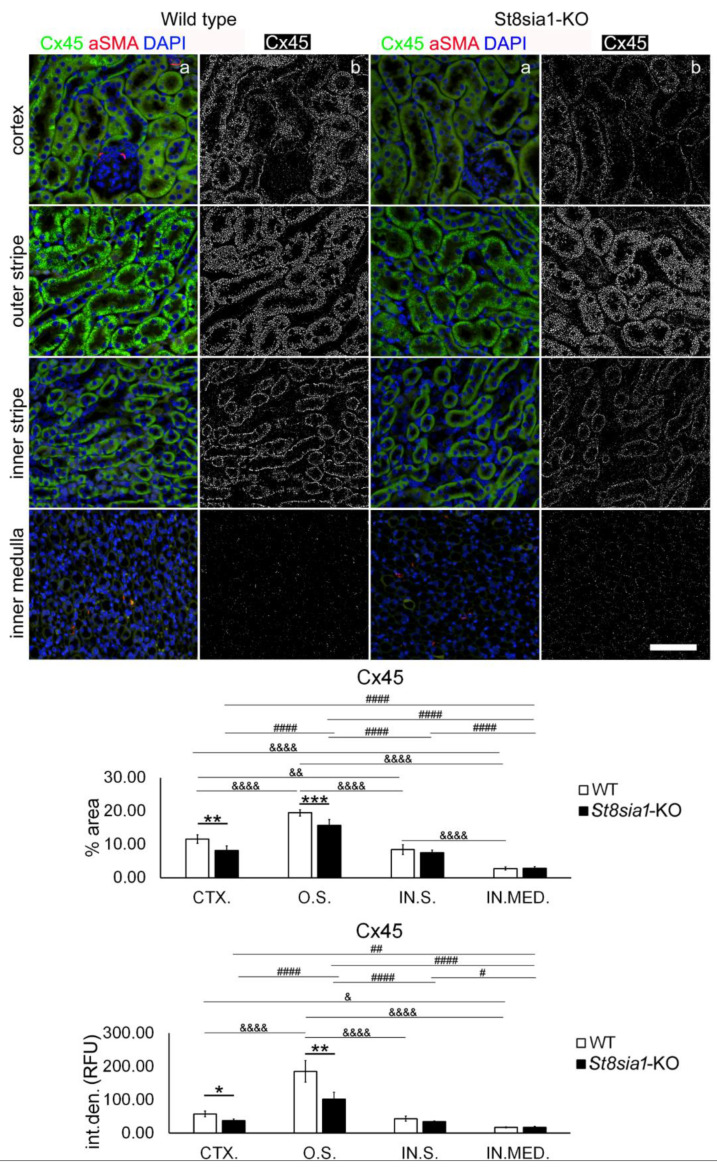
Expression of connexin 45 in different renal parts of the wild type and *St8sia1*-KO mice. WT—wild-type mice; *St8sia1*-KO—*St8sia1* knockout mice; CTX.—Cortex; O.S.—outer stripe of outer medulla; IN.S.—inner stripe of outer medulla; IN.MED.—inner medulla; % area—percentage of the section area occupied by Cx45 immunofluorescence; int.den. (RFU)—integrated density (relative fluorescence units). Column a—representative photomicrographs of the indicated kidney section areas stained with Cx45 (green) and alpha-smooth-actin (red) antibodies; nuclei are stained blue. Column b—(same section as in a) processed and thresholded photomicrographs prepared for analysis (inverted black for white on thresholded green image). Statistically significant difference between WT and *St8sia1*-KO: * *p* < 0.05; ** *p* < 0.01; *** *p* < 0.001 (*t*-test for unequal variances). Comparison between different kidney areas in the same group of mice WT: & *p* < 0.05, && *p* < 0.01, &&&& *p* < 0.0001; or *St8Sia1*-#—*p* < 0.05. ## *p* < 0.01, #### *p* < 0.0001 (one-way ANOVA followed by Tukey’s test for dependent samples). Scale-bar = 20 µm, refers to all (magnification 400×).

Similarly to Cx37, the expression of Cx45 was found to be abundant in all tubular structures in the cortex and inner and outer medulla. It was also found in the glomeruli of both WT and *St8sia1*-KO mouse kidneys, although the greatest density appeared to be in the proximal tubules (Figure 6 and Figure 7). Expression of Cx45 was highest in the medulla-particularly in the AQP2 immunoreactive cells of the collecting ducts and the AQP1 non-immunoreactive cells of the thick ascending limb. Expression was significantly lower in the PTCs and rare in the DTCs (DBA-positive) and the thin descending limb (AQP1 immunoreactive) (Figure 6). Analysis revealed significantly lower Cx45% area (*p* = 0.0045) and I.D. (*p* = 0.0114) in the CTX; and O.S. (% area *p* = 0.0007; I.D. *p* = 0.0082) in the *St8sia1*-KO mice, compared with the WT. Cx45% area and I.D. varied significantly between the different parts of the kidney sections, being highest in the O.S. and lowest in the IN.MED. (*p* from 0.0151 to *p* < 0.0001 compared with other parts of the kidney sections) in both WT and *St8sia1*-KO mice (Figure 7).

### 2.5. Expression of Pannexin 1 in the Kidney of Wild-Type and St8sia1-KO Mice

**Figure 8:**
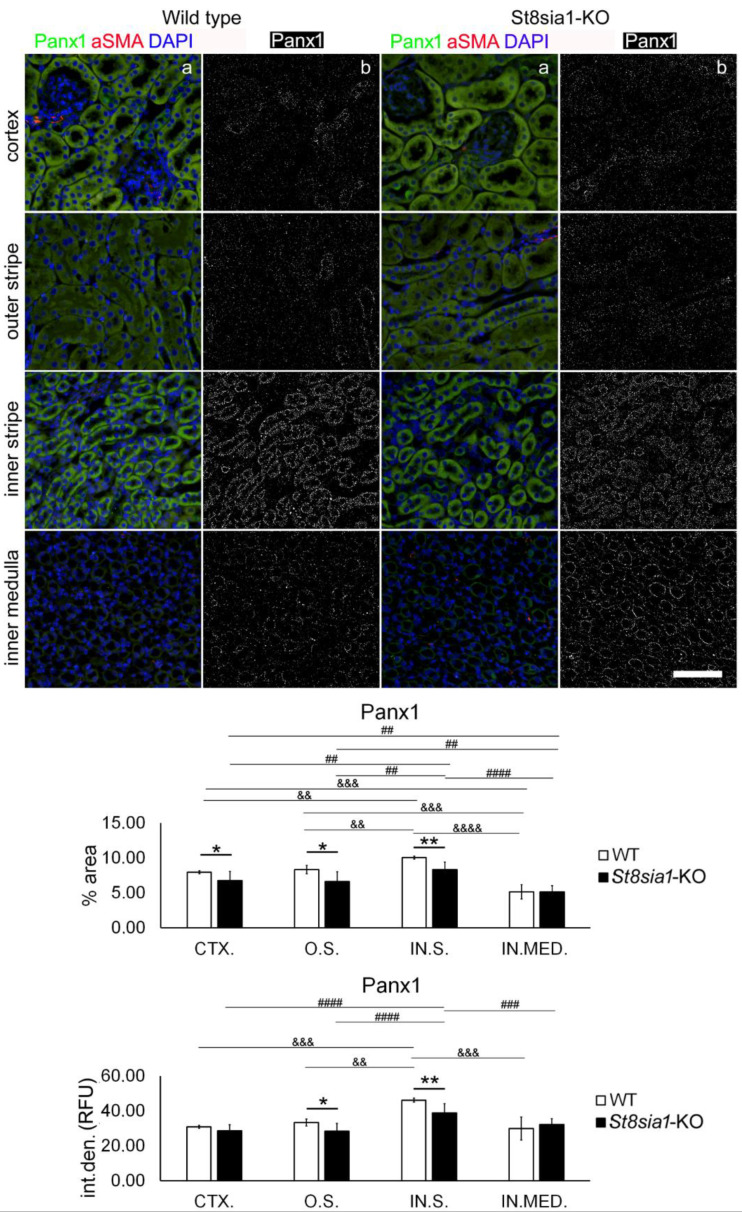
Expression of pannexin 1 in different renal parts of the wild type and *St8sia1*-KO mice. WT—wild-type mice; *St8sia1*-KO—*St8sia1* knockout mice; CTX.—Cortex; O.S.—outer stripe of outer medulla; IN.S.—inner stripe of outer medulla; IN.MED.—inner medulla; % area—percentage of the section area occupied by Panx1 immunofluorescence; int.den. (RFU)—integrated density (relative fluorescence units). Column a—representative photomicrographs of the indicated kidney sections stained with Panx1 (green) and alpha-smooth actin (red) antibodies; nuclei are stained in blue. Column b—(same section as a) processed and thresholded photomicrographs prepared for analysis (inverted black for white on thresholded green image). Statistically significant difference between WT and *St8sia1*-KO: * *p* < 0.05; ** *p* < 0.01; (*t*-test for unequal variances). Comparison between different kidney areas in the same group of mice WT: && *p* < 0.01, &&& *p* < 0.001, &&&& *p* < 0.0001; or *St8Sia1*-## *p* < 0.01. ### *p* < 0.001, #### *p* < 0.0001 (one-way ANOVA followed by Tukey’s test for dependent samples). Scale-bar = 20 µm, refers to all (magnification 400×).

We found expression of Panx1 in almost all tubular structures of the renal cortex and medulla in both strains of mice. However, in contrast to connexin, the most extensive Panx1 expression was observed in the IN.S. (Figure 8). Panx1 immunoreactivity was also found in the glomeruli. Double immunohistochemistry showed that Panx1 was most abundantly expressed in the AQP2-immunoreactive cells of the collecting ducts and was also conspicuous in the epithelium of the thick ascending limb (AQP-nonimmunoreactive). It was rare in the AQP1-immunoreactive epithelium of the thin descending limb (Figure 6). Significantly lower Panx1 expression (% area—*p* = 0.0394) was found in the CTX of *St8sia1*-KO mice compared with WT and in the O.S. (% area—*p* = 0.0144; I.D.—*p* = 0.0245). The largest difference was found in the IN.S. (% area—*p* = 0.0028; I.D.—*p* = 0.0048), whereas for the IN.MED., there was no significant difference in the expression of Panx1 or any of the connexins examined (Figure 8). In addition, Panx1 expression varied significantly between the different parts of the kidney sections. Panx1% area and I.D. were highest in IN.S. and significantly higher compared with all other parts of the kidney section (*p* from 0.0099 to *p* < 0.0001) in both WT and *St8sia1*-KO mice. Panx1 expression was lowest—Panx1% area was significantly lower compared with all other areas (*p* from 0.0040 to *p* < 0.0001), whereas Panx1 I.D. was significantly lower in IN.MED. compared with IN.S. in WT (*p* = 0.0005) and *St8sia1*-KO mice (*p* = 0.0008).

## 3. Discussion

In this study, we found immunoreactivity of all Cx studied in most tubular structures in the CTX. and IN.MED. and outer medulla, as well as in the glomeruli of both WT and *St8sia1*- KO mouse kidneys.

Although we found no differences between the different parts of the kidney section in terms of the percentage area occupied by Cx37 immunofluorescence, the highest Cx37 intensity of fluorescence was found in the IN.MED. The IN.MED. of the adult mouse kidney contains thin descending and thin ascending legs of the loop of Henle, collecting ducts, and blood vessels [46]. We found the strongest expression of Cx37 in the descending thin and the thick ascending limbs of the loop of Henle, while it was rare in the collecting ducts. The strong Cx37 staining intensity in the IN.MED. originated from its expression in the epithelium of the loop of Henle. As Cx37 was sparse in the collecting ducts, which also form the IN.MED., the percent area of immunofluorescence was not greater compared with the other parts of the renal section. We found abundant expression of Cx37 in the glomeruli and in the epithelial cells of the proximal and distal tubules, although it was less pronounced compared with expression in the epithelium of the loop of Henle. These results are consistent with previous findings in mice and rats that indicated strong basolateral expression of Cx37 in the epithelium of the thick ascending limb of the loop of Henle and in the distal tubule, whereas Cx37 expression was less pronounced in the proximal tubules and collecting ducts [1,47]. Gap junctions containing different connexin types are present in all glomerular cells [1]. Our results are in partial agreement with previous studies that demonstrated Cx37 expression only in mesangial cells of the vascular glomerular pole [1,48]. In support, a recent study in mouse animals detected Cx37 mRNA in the vascular endothelium and smooth muscle cells, in the endothelium of the glomerular capillary tuft localized around the vascular pole of the glomeruli, and in the medullary rays [49]. However, in contrast to our results, the authors of the aforementioned study did not detect expression of Cx37 mRNA in any tubule segment [49]. These discrepant results could be due to limitations of the study technique, as mRNA expression could be below the detection limit of RNAscope but still lead to protein synthesis.

Despite the strong expression of Cx37 in the IN.MED., there was no difference between the *St8sia1*-KO and WT mouse kidney in this part of the kidney section. However, the expression of Cx37 was lower in the CTX. and in both the O.S. and IN.S. of the outer medulla of the *St8sia1*-KO mice compared with WT mice. The renal CTX. contains glomeruli, proximal and distal tubules, connecting tubules, and collecting ducts. The thick ascending members of the loop of Henle and the pars recta, in addition to collecting ducts, are mainly located in the O.S., while the IN.S. contains thin descending members and thick ascending members of the loop of Henle and the collecting ducts [46]. In view of the observed distribution of Cx37 in the different tubule segments, we can conclude that the disruption of ganglioside composition caused by the absence of GD3 synthase has the greatest impact on the expression/trafficking of Cx37 in the proximal and distal tubules and the thick ascending limb. 

Renal expression of Cx40 was less pronounced compared with expression of Cx37. It was found in the glomeruli, DTCs, and thin descending limb. Rarer but distinct Cx40 puncta were clearly found in cells of collecting ducts. However, the most abundant expression of Cx40 was found in the PTCs and cells of the thick ascending limb in the medulla. Our finding of Cx40 immunofluorescence in the glomeruli is consistent with previous data by Hwan Seul and Beyer [50], who found abundant expression of Cx40 in the endothelium of the glomerular tuft. In addition, Zhang et al. [48] found Cx40 expression in mesangial cells throughout the glomerular area, although they did not find Cx40 in the glomerular endothelium. In agreement with our study, Silverstein and coworkers found Cx40 mRNA expression in the proximal and distal nephron using in situ RT-PCR, and they found that Cx40 mRNA expression increased 12 days after unilateral ureteral obstruction (UUO) in neonatal rats [51]. However, in contrast to our results, Cx40 mRNA was not found in rat glomeruli in the same study [51]. In support of our findings, Kundu and coworkers also found glomerular and tubular expression of Cx40 increased in the kidneys of diabetic AKITA mice [52]. A recent study in mouse kidney revealed Cx40 mRNA expression in the endothelium of blood vessels (intraglomerular endothelial cells at the glomerular hilum), glomeruli and medullary rays. In the glomeruli, coexpression of Cx40 mRNA and Platelet-derived growth factor receptor (PDGFR) mRNA indicated expression of Cx40 mRNA in mesangial cells, but the authors found no expression of Cx40 mRNA in any of the tubule segments. Similar to the results of the present study in mice, we previously found a pronounced expression of Cx40 in rat proximal tubules [53]. 

In our study, we found no difference in Cx40 expression between the kidney parts studied in WT mice. However, the lowest Cx40 percentage of area (% area) was found in the IN.MED. in *St8sia1*-KO mice. This difference is likely the result of decreased expression in the thin limbs of the loop of Henle, because we found few Cx40 puncta in the collecting ducts. However, we also cannot exclude a change in Cx40 expression in the endothelium of the peritubular capillaries in the IN.MED. In addition, the results of our study showed that the absence of GD3 synthase and disruption of ganglioside composition in the *St8sia1*-KO mice resulted in decreased Cx40 expression exclusively in the O.S. As we mentioned earlier, the O.S. contains mainly straight portions (S3 segment) of the proximal tubules and thick ascending members of the loop of Henle, as well as the collecting ducts [46]. Since we found abundant expression of Cx40 in both proximal tubules and thick ascending limbs, we can conclude that expression of Cx40 in these parts of the nephron is most sensitive to changes in ganglioside composition.

Expression of Cx43 was lowest compared with all other connexins examined. In agreement with our results, a recent study also found a weaker Cx43 mRNA signal compared to other connexins (Cx37, 40, and 45) in mouse kidney [49]. However, Cx43 mRNA expression in the mentioned study was restricted only to the interstitial capillary endothelium and the authors could not find expression of Cx43 mRNA in any tubular segment or glomerular cells [49]. We found the presence of Cx43 in glomeruli, PTCs, DTCs, and collecting duct cells. Moreover, Cx43 was most abundant in the thin descending limbs, whereas in contrast to all other connexins and Px1 examined, Cx43-immunoreactive puncta were rare (if not absent) in the cells of the thick ascending limbs. Our results are consistent with those of Kundu and coworkers, who also found increased glomerular and tubular expression of Cx43 in the kidneys of diabetic AKITA mice [52]. Previous studies have demonstrated the expression of Cx43 in the glomerular capillary tuft and in human podocytes, where they are the major components of the podocyte–podocyte connecting gap junctions [1,54]. However, in some studies, Cx43 expression was also not found in the glomerulus [48]. As for tubular expression, data are somewhat conflicting regarding expression in medullary collecting ducts [1,48,55,56,57]. In our study, we observed immunoreactive Cx43 puncta in both cortical and medullary collecting ducts. Similar to our and the above results in mice [52], the expression of Cx43 was also detected in proximal tubules of human kidney [1,58,59]. The results of our mentioned study in rats also confirmed the expression of Cx43 in proximal tubules [53]. However, in *St8sia1*-KO mice, the lowest Cx43% area was found in the IN.MED., but there was no difference in Cx43 expression between *St8sia1*-KO and WT mice in any of the areas examined. 

Expression of Cx45 was abundant in glomeruli and tubular structures in CTX. and medulla, with greatest density in collecting ducts and the thick ascending limb, lower in PTCs, and rare in DTCs and the thin descending limb of the loop of Henle. Our finding of Cx45 in glomeruli is consistent with the results of previous studies [1]. It has been suggested that Cx45 is expressed by podocytes and mesangial cells [60,61]. Data on Cx45 expression in distal tubules are conflicting, possibly due to species differences [1,51,57]. However, Cx45 expression was found in the proximal nephron of rat kidney [51]. In addition, our previous studies have revealed consistent expression of Cx45 in proximal tubules of mouse and rat kidneys [53,62,63]. 

The expression of Cx45 varied between different parts of the kidney sections, being highest in the O.S. of the outer medulla and lowest in the IN.MED., in both WT and *St8sia1*-KO mice. Since the O.S. contains mainly straight portions of proximal tubules and thick ascending limbs of the loop of Henle [46], this probably reflects the finding that the highest Cx45 expression was found in the thick ascending limb. On the other hand, because the IN.MED. consists of thin ascending and descending limbs, collecting ducts, and the peritubular capillaries [46], the lowest Cx45 density in this part of the kidney reflects our finding that the rarest Cx45-immunoreactive puncta were found in the thin descending limb. 

In the *St8sia1*- KO mice, Cx45 expression was decreased in the CTX. and the O.S. As these two areas contain mainly proximal tubules, we can assume that this decrease reflects the change in this particular tubule segment, in addition to the thick ascending limb in the O.S.

In the CTX., Panx1 immunoreactivity was found in the glomeruli, proximal and distal tubular epithelial cells, and collecting ducts. In contrast to the connexins, the most extensive Panx1 expression was found in the IN.S., reflecting the most abundant expression in the AQP2-immunoreactive cells of the collecting ducts and in the epithelium of the thick ascending limb. However, Panx1 expression was lowest in the IN.MED., reflecting rare findings in the AQP1-immunoreactive epithelium of the thin descending limb. The cell-specific localization of Panx1 expression in the mouse kidney studied by Hanner et al. is consistent with our results, except for Panx1 signaling in the glomerulus, which they failed to detect [64]. Another difference is that we found only rare Panx1-immunoreactive puncta in the AQP1-immunoreactive epithelium of the thin descending limb. 

The absence of GD3 synthase in the *St8sia1*-KO mice and the resulting ganglioside disruption resulted in lower Panx1 expression in the renal CTX. and O.S., presumably reflecting the decrease in the proximal tubules and collecting ducts. However, the greatest decrease in Panx1 expression was seen in the IN.S., likely reflecting a decrease in collecting ducts and the thick ascending limb. 

As an important component of intercellular signaling, Panx1 is involved in paracrine signaling by releasing ATP from cells through Panx1-constituted channels [65,66,67]. It is involved in several metabolic disorders and contributes to the pathophysiology of type 2 diabetes, as its increased expression in adipose tissue correlates with the degree of insulin resistance [27,68,69]. Panx1 has also been found to regulate the release of chemoattractants for phagocytic cells during apoptosis, while being dispensable for inflammasome activation [70]. Panx1 channels are thought to play an important role in regulating salt and water transport in the renal tubules, and thus, body fluid homeostasis, due to their ability to regulate ATP release into the tubule lumen and renal vasculature [64]. Altered ganglioside composition that alters the expression of Panx1 may affect the multiple functions of Panx1 in the kidney.

Here, we used mice lacking the GD3 synthase enzyme as a model to investigate possible changes in the expression of Cx and Panx1 due to a specific altered ganglioside composition. *St8sia1* KO mice are deficient in b-series (GD3, GD1b, GT1b) and c-series (GT3, GT2, GT1c) gangliosides and accumulate a-series gangliosides (GM1, GM2, GM3, and GD1a). As mentioned earlier, Cx channels in the plasma membrane are associated with lipid rafts and caveolae. The distribution of Cx channels in rafts and their channel function depend on the lipid composition and the specific biophysical properties of rafts [12,13,14,15,16,17]. Therefore, changes in the expression, distribution, and turnover of Cx are expected in lipid microenvironments with perturbed ganglioside composition and lipid raft properties. As we found, the expression of Cx37, Cx40, Cx45, and Panx1 was decreased in different parts of the kidneys of *St8sia1* KO mice compared with WT. The most consistent decrease was found in the O.S., where all markers (Cx 37, 40, 45, and Panx1) were disrupted in St8si1 KO mice; in the CTX., a decrease in the expression of Cx37, Cx45, and Panx1 was found, whereas in the IN.S., the expression of Cx37 and Panx1 was reduced.

The short lifespan of connexins includes their cotranslational insertion into the endoplasmic reticulum (ER) (where some of the newly synthesized Cx undergo ER -associated degradation), transport to the cell membrane in parallel with oligomerization to connexons, alternative docking to connexons from neighboring cells, or existence as hemichannels. In addition, they can form a connexosome by endocytosis, which is degraded by autophagy, or they can be recycled using the endosomal machinery [71]. Therefore, disruption of any step in these processes could lead to altered dynamics of cellular Cx synthesis and degradation, which in turn would result in an altered Cx profile. The changes in the expression of Cx that we found in the altered ganglioside microenvironment of the *St8sia1*-KO mouse kidney could be explained, at least in part, by the previously demonstrated influence of the ganglioside profile on autophagy. Autophagy is an essential homeostatic process by which cells degrade and recycle unnecessary or dysfunctional components and organelles, providing the cell with substantial energy resources. Impaired autophagy can lead to sphingolipid accumulation and contributes to the pathogenesis of some diseases [72,73,74]. Garofalo et al. reported the important role of GD3 ganglioside, a paradigmatic component of lipid rafts, in the formation of an autophagosome through mitochondria- ER crosstalk in the early events of the autophagy process. In their experiment, silenced expression of the *St8sia1* gene resulted in impeding autophagosome nucleation [32]. Thus, altered ganglioside composition in *St8sia1* KO mice could lead to impaired autophagy via changes in the content of lipid rafts. 

On the other hand, gap junction (GJ) channel degradation has been shown to be mediated by the clustering of Cx43 in lipid raft microdomains in COCs in cumulus-oocyte complexes [75]. Therefore, it might be expected that a change in lipid raft composition could lead to impaired GJ (and Cx) degradation. Although Panx proteins undergo similar pathways of synthesis, ER processing and hexamer assembly, and proteasome degradation of misfolded Panx proteins, unlike Cx, they have a longer half-life and do not utilize classical endocytotic machinery [22,76,77,78]. However, degradation of internalized Pxs is lysosomally dependent [77]. Therefore, the alteration in Panx1 expression that we found in *St8sia1* KO mice may also be related to a possible disruption of Panx1 synthesis, trafficking, and degradation. 

The most abundant renal ganglioside GM3 forms lipid rafts together with another ganglioside of the a-series GM1, in whose stability and formation sphingolipids play an important role [79,80]. In *St8sia1* KO mice, a-series gangliosides accumulate [36], and it is inevitable that the composition of gangliosides in lipid rafts changes. Lipid rafts act as platforms that coordinate signal transduction pathways in cells. Connexins are preferentially localized in lipid rafts, which may be involved in their redistribution between plasma membrane Cx hemichannels and GJ plaques. Recent evidence indicates that the distribution of Cx channels differs in rafts with different lipid compositions and that specific biophysical properties of rafts might influence Cx channel function [17]. This could be an explanation for the different “response” of the different Cx in the kidney to *St8sia1* KO observed in our study. For example, the level of Cx43 did not change in the kidney of *St8sia1* KO mice compared to WT, and Cx43 is known to preferentially caveolar Cx that are insoluble in Triton X-100 [15,81]. The regulation of Cx function by lipid rafts is also evident in tumor progression, where the involvement of Cx in cell migration/invasion depends on the localization of channels in lipid rafts such as caveolae [82]. Our data are consistent with previous suggestions that lipid rafts are key players in the dynamic redistribution and equilibrium of Cx between GJ plaques and plasma membrane hemichannels, and that different lipid rafts may also be involved in their intracellular trafficking process [17]. 

The main limitation of our study was that we used only immunohistochemistry as a method for quantification of different connexins in two strains of mice. However, fluorescence microscopy quantifies also a spatial information of protein distribution in tissue or cell in addition to fluorescence. The latter is crucial in our results because we also aimed to reveal a protein distribution in different areas of kidney (CTX., O.S. and IN.S. and IN.MED.) and different kidney structures, therefore, immunohistochemistry, which enables detection of spatial distribution, was chosen as the most adequate method. Although these enable quantification of proteins/gene products, with Western blot and qRT-PCR spatial resolution is lost, which was of great importance in our research.

## 4. Materials and Methods

### 4.1. Ethics

The experimental protocols and procedures complied with the Croatian Animal Welfare Act and ethical guidelines and were approved by the Regional Ethics Committee for Scientific Experiments.

### 4.2. Experimental Animals

Experiments were performed in twelve male 6-month-old mice. Four of them were wild-type control mice (C57BL/6-type, WT) and eight mice were *St8sia1* gene knockouts (*St8sia1* KO). The *St8Sia1* KO mice were originally obtained from The University of Missouri Mutant Mouse Resource & Research Center (*St8sia1*^tm1Rlp^). 

The animals were housed in self-ventilating cages (EHRET, Freiburg, Germany), with five air changes per minute, stable room temperature (21 ± 2 °C) and humidity (40–60%), and a 12/12 h light/dark cycle. They were fed standard laboratory chow (4RF21 GLP, Mucedola srl, Settimo Milanese, Italy).

### 4.3. Sample Collection and Preparation of Kidney Tissue for Immunohistochemistry

After administration of general anesthesia (Narketan^®^, 80 mg/kg + Xylapan^®^, 12 mg/kg, Vétoquinol, Bern, Switzerland), the mice were euthanized by decapitation. Kidneys were immediately removed and fixed by immersion in buffered 4% paraformaldehyde. During preparation for immunohistochemistry, tissues were washed in phosphate buffer saline (PBS, pH 7.2), then dehydrated in ethanol solutions of increasing concentrations (from 75% to 100%), cleared in xylene, and embedded in paraffin wax. Paraffin blocks were cut onto 5 μm thick slides using a microtome (Leica RM2155, Pittsburgh, PA, USA) and then mounted on glass slides. A standard deparaffinization procedure in xylene (three times for 5 min) was used, followed by rehydration of the tissue in ethanol solutions of decreasing concentration (2 times for 10 min in 100%, then once in 95%, and once in 70%) with a brief rinse with distilled water. Kidney sections were then heated in citrate buffer (pH 6.0) in a pressure cooker for 30 min for antigen recovery, cooled to room temperature, and washed with PBS.

### 4.4. Immunohistochemistry

After cooling to room temperature and washing in PBS, sections were covered with protein blocking solution (ab64226, Abcam, Cambridge, UK) for 20 min and then incubated overnight at room temperature with primary antibodies (Table 1). After incubation with the primary antibodies, samples were washed three times with PBS and incubated with the appropriate secondary antibodies for one hour at room temperature (Table 1). The samples were then washed in PBS. A two-minute DAPI (4′6-diamidino-2-phenylindole dihydrochloride) staining was performed to visualize the nuclei. After rinsing with distilled water, slides were air-dried and coverslipped (Immu-Mount, Shandon, Pittsburgh, PA, USA).

### 4.5. Data Acquisition and Analysis

Stained kidney sections were examined by immunofluorescence microscopy (BX61, Olympus, Tokyo, Japan) at 40× objective magnification and photographed with a cooled DS -Ri2 digital camera (Nikon, Tokyo, Japan) with NIS -Elements F software. To quantify connexin immunoexpression, nonoverlapping fields of view acquired at 40× magnification with constant exposure time were analyzed. Depending on the area of section size, 18–20 visual fields were acquired and analyzed for analysis of the cortex (CTX.); 6 for the outer stripe (O.S.); 3–4 for the inner stripe (IN.S.); and 2–4 for the inner medulla (IN.MED.). Green granular deposits were interpreted as positive Cx37, Cx40, Cx43, Cx45, and Panx1 immunoexpression. 

Quantitative evaluation of immunoreactivity was performed using ImageJ (National Institutes of Health, Bethesda, MD, USA). Images were prepared for analysis by subtracting the red counter signal from the green fluorescence. Then, the median filter with a radius of 7.0 pixels was applied and thresholded using a standard thresholding algorithm to obtain black and white 8-bit photographs. The percentage of area covered by Cx/panx immunofluorescence, and the integrated density were measured for each visual field, and an average value for all corresponding fields was used for statistical analysis.

Subtraction of background and contrasting was performed for the purpose of presentation.

### 4.6. Statistical Analysis

For statistical analyses, PAST 3.22 software (Øyvind Hammer, Natural History Museum, University of Oslo, Oslo, Norway) was used. Normality of the data was tested using the Shapiro–Wilk test. The difference between two groups was tested with the *t*-test for unequal variances. To compare different medullary and cortical parts of the section area, we used ANOVA followed by Tukey’s test for dependent samples. *p* < 0.05 was considered a statistically significant difference.

## 5. Conclusions

The results of the present study showed that deficiency of GD3 synthase in *St8sia1* KO mice leads to disturbance in renal Cx expression that is probably related to an alteration in ganglioside composition. The alteration was not observed for all Cx types examined in the different renal areas studied. Our data are consistent with the previously described role of lipid rafts in Cx distribution and transport. The observed disruption of Cx expression in the kidney of *St8sia1* KO mice may be one of the mechanisms for the previously found depletion of functional renal parenchyma caused by GM3 accumulation under hyperglycemic conditions.

## Figures and Tables

**Table 1:** Primary and secondary antibodies used.

	Antibody	Code No.	Host	Dilution	Source
Primary	Anti-Cx37/GJA4	ab181701	Rabbit	1:100	Abcam, Cambridge, UK
	Anti-Cx40/GJA5	ab213688	Rabbit	1:100	Abcam, Cambridge, UK
	Anti-Cx43/GJA1	ab87645	goat	1:100	Abcam, Cambridge, UK
	Anti-Connexin 45/GJA7/Cx45	ab135474	Rabbit	1:100	Abcam, Cambridge, UK
	Anti-pannexin 1/PANX1	ABN242	Rabbit	1:100	Merck KGaA, Darmstadt, Germany
	Anti-Aquaporin 1/AQP1 (B-11)	sc-25287	Mouse	1:50	Santa Cruz Biotechnology Inc., Santa Cruz, CA, USA
	Anti-Aquaporin 2/AQP2 (E-2)	sc-515770	Mouse	1:50	Santa Cruz Biotechnology Inc., Santa Cruz, CA, USA
	Anti-Smooth Muscle Actin	M0851	Mouse	1:300	Dako, Glostrup, Denmark
Lectins	Fluorescein labeled *Dolichos Biflorus* agglutinin (DBA)	FL-1031	-	1:400	Vector Laboratories Ltd., Peterborough, UK
	Fluorescein labeled Lotus Tetragonolobus lectin (LTL)	FL-1321	-	1:400	Vector Laboratories Ltd., Peterborough, UK
Secondary	Alexa Fluor^®^488 AffiniPure Anti-Goat lgG (H+L)	705-545-003	Donkey	1:400	Jackson Immuno Research Laboratories, Inc., Baltimore, PA, USA
	Alexa Fluor^®^488 AffiniPure Anti-Rabbit lgG (H+L)	711-545-152	Donkey	1:400	Jackson Immuno Research Laboratories, Inc., Baltimore, PA, USA
	Rhodamine Red™-X (RRX) AffiniPure Anti-Mouse IgG (H+L)	715-295-151	Donkey	1:400	Jackson Immuno Research Laboratories, Inc., Baltimore, PA, USA
	Rhodamine Red™-X (RRX) AffiniPure Donkey Anti-Rabbit IgG (H+L)	711-295-152	Donkey	1:400	Jackson Immuno Research Laboratories, Inc., Baltimore, PA, USA
	Rhodamine Red™-X (RRX) AffiniPure Anti-Goat IgG (H+L)	705-295-003	Donkey	1:400	Jackson Immuno Research Laboratories, Inc., Baltimore, PA, USA

## Data Availability

Data related to this study are available upon reasonable request from the corresponding author.
